# Potential legal issues arising from the use of artificial intelligence in ophthalmic diagnosis

**DOI:** 10.1177/00258172251347372

**Published:** 2025-11-19

**Authors:** Edward Burton

**Affiliations:** Royal Holloway, University of London, London, UK

**Keywords:** AI, healthcare, ophthalmology, responsiblity, legal issues

## Abstract

Artificial intelligence (AI) in health care can assist with accurate detection of retinal diseases. However, UK Law does not currently explicitly provide for its regulation or liability for errors caused by an artificial intelligence system but those who use it may be held responsible when it malfunctions and causes harm. Who should be held liable when an AI image production or diagnostic system causes error? The system itself and those who produce it or the operators?

## Introduction

AI has great potential to improve health care – and one such area is ophthalmology. It can assist with the diagnosis of diseases, such as diabetic retinopathy and age-related macular degeneration.^1^ AI can also provide synthetic images generated by deep learning systems which can then be applied as an adaptive optics (AO) learning method, which can be used to detect aberrations disrupting the camera’s view and generate clear images of retinal structures.^2,3^ There is considerable potential for AI use in ophthalmology, however, AI’s current uncertain legal status in terms of who will be held responsible if harm results from its malfunctions or errors may limit enthusiasm for its wider application.

This technology, along with others, requires further discussion about its legal status as well as its ethical application.

## Relevant uses of artificial intelligence in ophthalmology

Are the benefits of AI sufficient to justify changes to the law? There has already been extensive debate, and more is required if the law is to be altered. Below are three examples of AI systems that are currently in use.

### Adaptive optics

Adaptive optics systems offer an increasing number of clinical applications. As light is shone into the eye, a wavefront sensor detects aberrations in the reflected light, which enables an ophthalmic device, such as conventional fundus imaging, to adjust the shape of the light, so as to minimise aberrations and procure a clearer image.^4^

### Detection of diabetic retinopathy

AI can accurately detect diabetic retinopathy and retinal diseases. A study performed in 2023 by EyeArt, a part of the American Academy of Ophthalmology, compared the performance of an AI screening system with human ophthalmologists. The results showed that the AI screening system was more sensitive to the detection of more than mild diabetic retinopathy (mtmDR) than when performed by general ophthalmologists and retina specialists.^5^ This corresponds with the 2016 test of the IDx-DR X2.1 AI software, which also detected signs of non-proliferative diabetic retinopathy, specifically identifying dot blood haemorrhages, hard exudates and neovascularisation, with extremely high accuracy.^6^

### Detection of glaucoma

There have been several studies of the use of machine learning systems to detect glaucoma in its early stages in recent years. Yousefi et al.^7^ found in 2022 that an unsupervised, automated machine learning system could detect patterns of visual field loss associated with the early stages of glaucoma. AI systems have been proven to be highly accurate in diagnosing glaucoma based on fundus photography; 96.2%. In another 2022 study, however the authors, Chaurasia et al.,^8^ claim that its performance was ‘unrealistically excellent’ and so was unsuitable for clinical implementation at the time. This suggests that an absence of legal regulation is not the sole reason that AI systems are not widely used in glaucoma diagnosis, rather, that a lack of confidence in the technology itself is also preventing its implementation.

## Current and proposed regulation

At the moment, no statute has been passed for the specific purpose of regulating the use of AI in health care. The legislation currently used for this is the Medical Devices and Regulations 2002 and the Medicines and Medical Devices Act 2021.

The Medicines and Healthcare products Regulatory Agency (MHRA) is at present the regulatory authority with jurisdiction over AI. However, a Private Members’ Bill is currently under scrutiny in the House of Lords regarding the regulation of AI in the UK: the Artificial Intelligence (Regulation) Bill.^9^ This is contradictory to the government’s previous position on AI regulation, as noted in the library briefing of the Bill itself: ‘it [the government] contends it is too soon in these technologies’ evolution to legislate effectively and to do so now may be counterproductive’. The Bill comprises nine clauses. Clauses 1, 2, 3 and 8 are particularly notable. Clause 1 would delegate to the Secretary of State the competence to create an AI Authority. Clauses 2 and 3 would set out the principles which would be considered by the AI Authority in its regulation of artificial intelligence. Clause 8 would provide for the creation of offences, liability for which would arise from breach of the regulations set out in clauses 2 and 3.

The Artificial Intelligence (Regulation) Bill would replace the March 2023 AI White Paper, which was criticised for a variety of reasons. The University of Bristol Law School noted its perceived lack of detail regarding the meaning of its principles,^10^ and the Ada Lovelace Institute expressed concern that it did not create legal obligations, instead relying on ‘voluntary commitments to good practice’, which could not ensure compliance with the regulations.^11^ This exemplifies the lengthy process of debate and amendments required before enforcement, which may be detrimental to medical institutions seeking to use, or already using, artificial intelligence, as it is unclear what regulations will be enforced and whether or when any conclusion at all will be reached, as the Bill has not yet reached the House of Commons. This risks a similar outcome to the Canadian Artificial Intelligence and Data Act, which was tabled in 2022, and still shows no indication of being enforced in the near future.^12^ An ethical dilemma is raised: should attempts to accelerate the process to enforce regulations on AI in health care be avoided as it could result in a poorly constructed, inefficient authority, or should they be pursued so that patients with degenerative diseases can be diagnosed with a lower margin of error than human ophthalmologists?

## The question of tort liability

A broader ethical issue arises from the increasing use of AI, which is whether a misdiagnosis made by a deep learning system would create liability for a clinical negligence claim, or if the fault would be attributed to the system; and if the latter, the extent to which that would be. Following the Medicines and Medical Devices Act 2021, the relevant question in such a case would be whether a reasonable health professional would have relied on the information in the same way. This issue applies to health care as a whole but is particularly prevalent in ophthalmology, due to the high rate of misdiagnosis of neuro-ophthalmic conditions.^13^

Babushkina^14^ argues that a mistake fundamentally cannot be made by an AI system, only by its user, as any incorrect result is due to a mistake in syntax by its programmer. From this perspective, the issue is simplified somewhat, as the relevant claim would conclusively be tort in negligence against the creator of the system. Babushkina makes the reasonable point that an AI system cannot realistically be an object of blame in such cases. This has not, however, been enforced as due process by statute, nor has any comment on the matter been made by the MHRA. The manufacturer or supplier of the AI system may also be held responsible, if the machine is misrepresented as having superior capabilities than it possesses, or is inadequately maintained.

It could be argued that there is no need for reform, if the categorisation of deep learning system AI as a medical device as an extension of ‘Software as a Medical Device’ is truly accurate, but the international enforcement of AI regulations, and further propositions of the sort, suggest otherwise.^15^

## Conclusion

As the use of AI in health care is becoming more commonplace, the need for a statute and authority specifically dedicated to its regulation in the UK and worldwide is increasing. Ophthalmology is an area which would be particularly noticeably impacted by the implementation of deep learning and machine learning systems as AI systems have already proven to be extremely accurate in detection of retinal diseases in their early stages. It is unlikely that that the UK Parliament will pass any legislation soon to create an AI authority, as is also the case internationally.
